# Factors affecting hematological parameters in Creole goats on the Southern Coast of Peru

**DOI:** 10.14202/vetworld.2025.2002-2011

**Published:** 2025-07-22

**Authors:** Jhony Soca, Emmanuel Alexander Sessarego, Pamela Sarmiento, María José Cevallos-Cardenas, Juan Canchino-Gutierrez, Jose Teran, Jose Antonio Ruiz, Juancarlos Cruz-Luis, Danny Julio Cruz

**Affiliations:** 1 Estación Experimental Agraria Chincha, Dirección de Servicios Estratégicos Agrarios, Instituto Nacional de Innovación Agraria (INIA), Ica 11770 Peru; 2 Dirección de Servicios Estratégicos Agrarios, Instituto Nacional de Innovación Agraria (INIA), Lima 15024, Peru; 3 Escuela de Medicina Veterinaria y Zootecnia, Universidad Privada San Juan Bautista, Ica 11004, Peru; 4 Departamento de Producción Animal, Facultad de Agronomía, Universidad de Buenos Aires, Buenos Aires C1417, Argentina

**Keywords:** body condition score, creole goat, extensive production system, feeding regimen, hematological parameters, Peru, robust regression

## Abstract

**Background and Aim:**

Hematological parameters are critical indicators of health and physiological status in goats. This study aimed to evaluate the effects of location, feeding regimen, age, and body condition score (BCS) on hematological parameters in Creole goats reared under extensive systems on the southern coast of Peru and to establish context-specific reference values.

**Materials and Methods:**

A total of 111 multiparous goats from nine herds were assessed. Red blood cell (RBC) (RBC, hematocrit, hemoglobin [HGB], mean corpuscular volume [MCV], mean corpuscular hemoglobin [MCH], and MCH concentration [MCHC]) and white blood cell (WBC) (WBC, lymphocytes, monocytes [MON], neutrophils [NEU], and eosinophils [EOS]) parameters were determined using a veterinary hematology analyzer. Robust linear regression models with MM-estimation were applied, with model selection based on Akaike information criterion, Bayesian information criterion, and root mean square error. Correlation analyses and hierarchical clustering were also performed to explore inter-parameter relationships.

**Results:**

Significant interindividual variation was noted, particularly among leukocyte indices (EOS, MON, NEU; coefficient of variation >50%). In contrast, MCH and MCV exhibited low variability. The geographic location was not statistically significant, suggesting environmental homogeneity across the sites. Dietary composition and BCS significantly influenced several hematological indices. Goats with higher BCS and mixed alfalfa-residue diets exhibited increased HGB and RBC counts, whereas younger goats showed higher MCHC values. Predictive equations were generated to estimate hematological values under specific management conditions.

**Conclusion:**

This study highlights the relevance of age, nutritional status, and body condition in modulating hematological values in Creole goats. The derived models and reference values can inform localized diagnostic criteria and enhance decision-making in goat health management under extensive systems. Future studies should incorporate seasonal, sex-based, and longitudinal analyses to refine predictive accuracy.

## INTRODUCTION

Goats are among the principal livestock species globally, especially in regions where their resilience and low maintenance requirements make them ideal for smallholder farming systems [1]. Additionally, they significantly contribute to rural economies by enha-ncing food security and supporting the livelihoods of numerous families [2]. Their adaptability to various production systems has enabled their successful mana-gement under conditions ranging from intensive to ext-ensive systems [3].

In the southern coastal region of Peru, Creole goats are predominantly managed under extensive systems characterized by natural grazing, minimal reliance on external inputs, and relatively low productivity [4]. These goats are well adapted to coastal environments due to favorable climatic conditions. As of 2021, the national goat population was estimated at approximately 1,801,257 animals, with 4.17% located in the Ica region [5]. Despite their importance to regional econo-mies and food systems, limited research has been conducted to explore the health and nutritional status of Creole goats raised under extensive management. This knowledge gap hinders the development of targeted management and health strategies aimed at improving productivity and welfare.

Hematological evaluations are practical diagnostic tools for assessing the physiological, metabolic, and immune status of livestock [6]. They also support the diagnosis of diseases and the early detection of abn-ormalities caused by infections or environmental stress-ors [7]. However, accurate interpretation requires population-specific reference intervals tailored to the production system and prevailing environmental cond-itions [8].

These hematological parameters reflect the functional status of both the circulatory and immune systems [9]. The red cell series includes red blood cell (RBC) count, hematocrit (HCT), hemoglobin (HGB), mean corpuscular volume (MCV), mean corpuscular hem-oglobin (MCH), and MCH concentration (MCHC). The white blood cell series comprises the total white blood cell (WBC) count and its subtypes: lymphocytes (LYN), monocytes (MON), neutrophils (NEU), eosinophils (EOS), and granulocytes. In addition, platelet indices include platelet count and mean platelet volume [10, 11].

Hematological variation may be influenced by factors such as breed, age, nutritional status, physi-ological condition, altitude, season, reproductive stage, and management practices [12, 13]. In Peruvian Creole goats, these variations may be more pronounced due to the unique environmental conditions and extensive management practices, as well as inconsistent feed ava-ilability. Nonetheless, empirical data on hemat-ological indices specific to this population remain scarce, complicating clinical and productive assessments.

Despite the economic and nutritional importance of Creole goats in the southern coastal regions of Peru, limited scientific attention has been given to their physiological and hematological profiles under extensive management systems. Existing studies on hematological parameters in goats predominantly focus on standardized or exotic breeds raised under semi-intensive or intensive systems, primarily in temperate or controlled environments. These findings are not directly applicable to Creole goats, which are managed in environments characterized by low-input systems, seasonal forage variability, and diverse agroecological stressors. Moreover, factors such as body condition, age, and nutritional regimen – known to significantly influence hematological indices - remain underexplored in the context of Peruvian Creole goats. In particular, there is a scarcity of population-specific reference intervals and predictive models that account for these physiological and management-related variables. This gap hampers effective health monitoring, disease diagnosis, and formulation of nutritional strategies for goats reared in extensive systems.

This study aims to evaluate the hematological profiles of multiparous Peruvian Creole goats raised under extensive production systems on the southern coast of Peru. Specifically, it seeks to determine the influence of key variables, namely, feeding type, body condition score (BCS), age, and geographic locat-ion, on red and white blood cell parameters using robust statistical modeling. By generating localized reference values and identifying significant predictors of hematological variation, this research intends to support the development of evidence-based mana-gement strategies for health assessment, disease surveillance, and productivity optimization in Creole goat populations. The findings are expected to improve diagnostic accuracy and inform breeding and nutritio-nal interventions tailored to extensive goat-rearing systems in similar ecological zones.

## MATERIALS AND METHODS

### Ethical approval

All procedures were approved by the Institutional Ethics Committee of Universidad Privada San Juan Baus-tista (Resolution 0831-2024-CIEI-UPSJB) in compliance with animal welfare and ethical research standards.

### Study period and location

This study was conducted in early spring (October 2024) in two districts of the Ica region, southern Peru: Independencia (Latitude: 13° 38’ 49” S; Longitude: 76° 0’ 47” W; altitude: 211 m) and San Clemente (Latitude: 13° 38’ 32” S; Longitude: 76° 8’ 35” W; altitude: 76 m). Both sites are situated in a coastal desert climate, with an annual precipitation of approximately 25 mm and temperatures ranging from 14°C to 25°C. San Clemente experiences a slightly higher average relative humidity (70% vs. 62%), reduced irrigation access, and longer walking distances to grazing areas. These districts represent the highest concentrations of goat livestock within the Ica region [5].

### Production systems and animal management

Goat rearing in both Independencia and San Clemente follows an extensive production model. Re- productive cycles are seasonally defined, with mating typically occurring between October and December and kidding from March to May. The primary objective of rearing is milk production, which generally continues until the fourth parity, or in exceptional cases, the fifth.

Some producers practice limited transhumance, providing temporary access to natural pastures and native shrubs such as *Prosopis pallida* (huarango). However, alfalfa (*Medicago sativa*) constitutes the main dietary component in both districts. This forage is often supplemented with agricultural residues (e.g., corn, cassava, beans, cotton, and sweet potato), a practice more prevalent in San Clemente.

Animal care involves daily daytime grazing and nighttime housing in backyard corrals. Annual dewo-rming protocols are implemented postpartum using oral anthelmintics such as triclabendazole, albendazole, and mebendazole.

### Animal selection and nutritional background

A total of 111 multiparous Creole goats were selected through non-probability sampling, based on the accessibility and willingness of farmers to parti- cipate. All animals had undergone at least one par-turition and were in various stages of lactation, with an average body weight of 41.00 ± 2.1 kg. Of these, 66 goats originated from five herds in San Clemente and 45 from four herds in Independencia.

Two distinct feeding regimens were followed: Exclusive alfalfa or a mixed diet of alfalfa and crop residues. Alfalfa is a high-protein forage (up to 18%) with elevated β-carotene and metabolizable energy ranging from 2.1 to 2.4 Mcal/kg dry matter (DM). It also provides 40%–45% neutral detergent fiber (NDF), which supports ruminal function [14]. Agricultural by-products exhibited broader nutrient variability (protein: 4%–25%; energy: 1.6–3.2 Mcal/kg DM; NDF: >12%) [15, 16] and contributed dietary flavonoids, B vitamins, and iron. These compounds, especially flavonoids, are known for antioxidant properties that stabilize erythrocyte membranes and mitigate oxidative lipid damage [17, 18].

### Body condition scoring and health assessment

BCS were determined using the five-point Russell scale [19], based on palpation and visual assessment of muscle and fat deposition over anatomical land-marks such as the tail head, ribs, and spine. Coprological evaluations were conducted to screen for parasitism. Deworming was implemented using a rotation of tricla-bendazole (12 mg/kg) and fenbendazole (10 mg/kg), followed by a 45-day withdrawal period before blood collection to eliminate confounding pharmacological effects on hematological profiles.

### Blood sample collection

Blood was collected through jugular venipuncture between 07:00 h and 09:00 h using 20-gauge needles and 3 mL ethylenediaminetetraacetic acid (EDTA)-coated vacutainer tubes (BD Vacutainers, Becton Dickinson and Company, USA). Samples were immediately stored in a cooled container (4–8°C) and transported to the Animal Health Laboratory at the Faculty of Veterinary Medicine and Zootechnics, Universidad Nacional San Luis Gonzaga, for hematological analysis.

### Hematological analysis

Hematological parameters were measured using the veterinary analyzer “Animal 4-part Hematology GEN-VET VH-40 (Genvet, a brand of Melet – Schloesing Laboratories, France) calibrated for Caprine species. A 9 μL sample aliquot was used to quantify the following indices (WBC, %), (LYN, %), (MON, %), (NEU, %), (EOS, %), (RBC, 10¹²/L), (HGB, g/dL), (HCT, %), (MCV, fL), (MCH, pg), and (MCHC, g/dL).

Two methodologies were employed:

- Impedance method: Quantification of blood cells was based on changes in electrical resistance as cells passed through an aperture flanked by electrodes, with the magnitude of resistance proportional to cell size.
- Colorimetric method: Hemoglobin was quantified using a chromogenic reagent, and optical density was measured through spectrophotometry using the integrated optical system of the GEN-VET VH-40 (Genvet, a brand of Melet – Schloesing Laboratories, France).

### Statistical analysis

Descriptive statistics were calculated to summ-arize central tendencies and dispersion of hematol-ogical variables. Due to the presence of outliers and non-normal distributions, Spearman’s rank correlation coefficients (ρ-s) were computed to assess inter-parameter associations. Hierarchical clustering using the single linkage method was conducted, yielding a phenetic correlation coefficient of 0.89.

Robust linear regression models were developed to account for data irregularities. Models ranged from saturated (including all main effects and two-way interactions of locality, feeding regimen, age, and BCS) to simplified forms. Model selection was guided by the Akaike information criterion, Bayesian information criterion, and root mean square error, which prioritize goodness of fit while penalizing model complexity.

The MM-estimation method (from the *MASS* package, Modern Applied Statistics with S, version 7.3-64 - 2025.01-04, University of Oxford, Oxford, United Kingdom) was used to reduce the influence of outliers. MM-estimation is a two-step procedure involving M-estimators, which are parameter estimates obtained by minimizing a loss function of the residuals to limit the impact of extreme values. This approach estimates regression coefficients through weighted residual minimization, where a robust weight matrix adjusts the impact of observations based on the magn-itude of their deviations. Tukey’s bisquare function was applied for weight adjustment, preserving estimator efficiency under normality [20]. Pairwise comparisons were conducted using Tukey’s test within the *emmeans* package version 1.10.7 - 2025-01-31 (The University of Iowa, Iowa City, United States) [21]. All analyses were conducted using RStudio Desktop version 4.4.2 -2025.05.1+513 (R-Statistic, Vienna, Austria) [22].


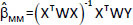


Where β^_MM_ represents a vector with estimated coefficients, X is the incidence matrix including the effects considered in each model, and Y contains the observed hematological parameter values. The robust weight matrix W = diag(w_i_) adjusts the contribution of each observation based on the influence function ψ(r_i_), which modifies the impact of standardized residuals where S is a robust estimate of residual dispersion:


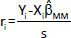


In this study, Tukey’s square function was used, which reduces the influence of outliers without compromising the estimator’s efficiency in normal distri-butions [23]. Multiple comparisons were performed using Tukey’s contrast in the means package [21]. All analyses were conducted using R Studio version 4.4.2 [22].

## RESULTS

### Descriptive variability in hematological parameters

The hematological profiles of Creole goats reared under extensive production systems exhibited consi-derable interindividual variability. The coefficient of variation across parameters ranged from 4.83% to 57.80%, as shown in Tables 1–3. The highest variation was observed in leukocyte indices, notably EOS (57.80%), MON (52.09%), and NEU (54.84%), indicating pronounced heterogeneity in immune response within the population. In contrast, erythrocyte indices such as MCH (4.83%) and MCV (6.87%) were the most stable, reflecting relatively uniform erythrocyte metrics.

**Table 1:** Descriptive analysis of hematological parameters in Creole goats under an extensive system.

Parameters	Mean	Median	SD	CV (%)	Minimum	Maximum
MCH (pg)	7.14	7.10	0.34	4.83	6.50	9.20
MCHC (g/dL)	37.52	36.70	3.28	8.75	31.80	55.30
MCV (fL)	19.11	19.00	1.31	6.87	16.60	22.80
HCT (%)	21.11	21.30	2.75	13.04	12.20	26.70
HGB (g/dL)	7.86	7.80	0.83	10.57	5.70	9.80
RBC (10¹²/L)	11.05	11.11	1.28	11.60	7.16	13.93
MON (%)	0.66	0.64	0.34	52.09	0.01	1.59
LYN (%)	3.39	3.13	1.32	39.02	1.15	7.83
EOS (%)	0.74	0.65	0.43	57.80	0.20	2.32
NEU (%)	5.01	4.47	2.75	54.84	1.02	18.86
WBC (%)	9.81	9.12	3.81	38.88	3.99	25.21

SD=Standard deviation, CV=Coefficient of variation, MCH=Mean corpuscular hemoglobin, MCHC=Mean corpuscular hemoglobin concentration, MCV=Mean corpuscular volume, HCT=Hematocrit, RBC=Red blood cell count, HGB=Hemoglobin, MON=Monocytes, LYN=Lymphocytes, EOS=Eosinophils, WBC=Total white blood cell count, NEU=Neutrophils.

**Table 2:** Estimated coefficients from robust regression and adjusted marginal means (MM-estimates) with standard errors for red blood cell parameters in Creole goats, according to feeding type, locality, age, and their relationship with body condition score (BCS), using 2.7 as the reference.

Variables	MCH (pg)	MCHC (g/dL)	MCV (fL)	HCT (%)	HGB (g/dL)	RBC (10¹²/L)
Intercept	7.10**	40.87	19.06	5.36	1.77	2.86
Feeding						
Alfalfa (ref)	-	-		-		
Alfalfa + residues	-	-	-0.62*	-	0.40**	0.48
Locality						
Independencia (ref)	-	-	-	-	-	-
San Clemente	-	-	-	-	-	-
Age (years)						
2 (ref)	-		-	-	-	-
3	-	-4.23*	-	-	-	-
4	-	-3.90*	-	-	-	-
>4	-	-4.57*	-	-	-	-
BCS	-	-	-	5.87**	2.16**	2.93**
AIC	83.28	586.33	378.57	533.23	265.69	365.60
BIC	88.58	591.64	383.88	541.14	276.15	373.51
RMSE	0.35	3.33	1.31	2.60	0.77	1.22
Mean	7.10	40.90^a^ 36.67^b^ 36.97^b^ 36.29^b^	19.50^a^ 18.90^b^	21.19	7.60^a^ 8.01^b^	10.80^a^ 11.20^a^
Standard Error	0.03	1.02 0.43 0.33 0.70	0.22 0.15	0.25	0.13 0.09	0.21 0.14

Ref: Reference. Different letters within each hematological parameter indicate statistically significant differences (p < 0.05);

* and

** indicate statistical significance at 95% (p < 0.05) and 99% (p < 0.01), respectively; AIC=Akaike Information Criterion, BIC=Bayesian Information Criterion, RMSE=Root Mean Square Error, MCH=Mean corpuscular hemoglobin, MCHC=Mean corpuscular hemoglobin concentration, MCV=Mean corpuscular volume, HCT=Hematocrit, RBC=Red blood cell count, HGB=Hemoglobin.

**Table 3:** Estimated coefficients from robust regression and adjusted marginal means (MM-estimates) with standard errors for white blood cell parameters in Creole goats, according to feeding type, locality, age, and their relationship with body condition score (BCS), using 2.7 as the reference.

Variables	MON (%)	LYN (%)	EOS (%)	NEU (%)	WBC (%)
Intercept	-0.98	3.32**	0.67**	4.16	8.73
Feeding					
Alfalfa (ref)	-	-	-		
Alfalfa + residues	-	-	-	0.50	0.86
Locality					
Independencia (ref)	-	-	-	-	-
San Clemente	-	-	-	-	-
Age (years)					
2 (ref)	-	-	-	-	-
3	-	-	-	-	-
4	-	-	-	-	-
>4	-	-	-	-	-
BCS	0.53**	-	-	-	-
AIC	80.57	380.82	133.40	546.64	617.29
BIC	85.88	386.13	138.69	551.94	622.60
RMSE	0.34	1.32	0.43	2.79	3.83
Mean	0.63	3.32	0.67	4.16^a^ 4.65^a^	8.73^a^ 9.59^a^
Standard Error	0.03	0.13	0.04	0.29 0.20	0.55 0.38

Ref: Reference. Different letters within each hematological parameter indicate statistically significant differences (p < 0.05);

** indicate statistical significance at 99% (p < 0.01), AIC=Akaike Information Criterion, BIC=Bayesian Information Criterion, RMSE=Root Mean Square Error, MON=Monocytes, LYN=Lymphocytes, EOS=Eosinophils, WBC=Total white blood cell count, NEU=Neutrophils.

Notably, the variability within WBC indices was substantial; for example, NEU counts reached levels up to 18.5 times higher than the minimum recorded value. In contrast, erythrocytic parameters, including HCT, HGB concentration, and MCHC, exhibited limited dispersion, suggesting more consistent values across animals.

### Correlation structure and cluster analysis

**Figure 1:**
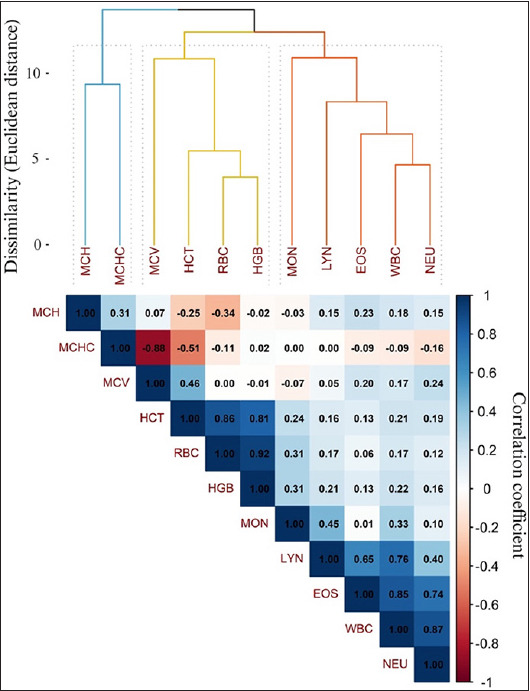
The Spearman correlation matrix œĂ and dendrogram of hematological parameters in Creole goats under an extensive system in Pisco Province, Ica region. Variables were grouped using hierarchical clustering based on Euclidean distance and single linkage. The dendrogram represents the dissimilarity between variables (1-ρ), with three clusters identified using the silhouette method. The triangular matrix displays correlation coefficients, with blue shades indicating positive correlations and red shades negative correlations, proportional to their magnitude. MCH=Mean corpuscular hemoglobin, MCHC=Mean corpu-scular hemogl-obin concentration, MCV=Mean corpuscular volume, HCT=Hematocrit, RBC=Red blood cell count, HGB=Hemoglobin, MON=Monocytes, LYN=Lymphocytes, EOS=Eosinophils, WBC=Total white blood cell count, NEU=Neutrophils.

Hierarchical clustering analysis based on Spearman correlation coefficients (ρ_s) revealed three distinct clusters of hematological variables (Figure 1).

- Cluster 1 included MCH and MCHC, showing a moderate positive correlation (ρ_s = 0.31).
- Cluster 2 consisted of RBC, HGB, HCT, and MCV, with strong correlations ranging from 0.81 to 0.92. Notably, MCHC exhibited negative correlations with both MCV and HCT, which explains its segregation from this group.
- Cluster 3 included WBC, NEU, LYN, and EOS, with intercorrelations spanning from 0.40 to 0.87. MON demonstrated moderate associations with LYN (ρ_s = 0.45) and WBC (ρ_s = 0.33).

### Effects of location, feeding, age, and BCS

Robust linear regression analysis determined that geographic location did not significantly contribute to hematological variation. As a result, location was excl-uded from the final predictive models. Table 2 presents the results for RBC parameters, while Table 3 outlines findings for white blood cell indices. These findings support the use of common reference values across the two study locations.

Intercept-only models, indicating no significant influence from age, feeding type, or BCS, were identified as optimal for MCH, LYN, and EOS. Conversely, significant predictors emerged for other parameters:

- HCT: Coefficient = 5.87
- RBC count: Coefficient = 2.93
- HGB concentration: Coefficient = 2.16
- MON count: Coefficient = 0.53 (all p < 0.01).

The feeding regimen also affected select variables. Goats receiving a mixed diet of alfalfa and crop residues exhibited a 3.05% decrease in MCV (p < 0.05) and a 3.70% increase in HGB (p < 0.01) compared to those fed exclusively on alfalfa (assuming a BCS of 2.7). While feeding type remained in the final models for RBC, WBC, and NEU, its effects in these cases were not statistically significant (p > 0.05). Age exerted a significant effect on MCHC, which was found to be 9.63%–11.27% higher in 2-year-old goats than in older individuals.

### Predictive modeling and reference value estimation

Predictive equations were developed based on the final robust models to estimate hematological values under specific combinations of BCS and feeding type. For instance, the HGB concentration for a goat with a BCS of 3 and fed either alfalfa alone or a mixed diet can be calculated as follows:

HGB = 1.77 + 2.16 (BCS) + 0.40 (FT)

Where:

- BCS
- FT = Feeding Type (0 = alfalfa only; 1 = alfalfa + crop residues).

For a goat with BCS = 3 and FT = 1:

HGB = 1.77 + 2.16 (3) + 0.40 (1) = 8.65 g/dL

Similar predictive estimates can be derived for other parameters showing statistically significant model coefficients. The marginal means obtained through the MM-estimation method (Tables 2 and 3), adjusted for a standard BCS of 2.7, were generally closer to the observed medians than to the means reported in Table 1. This underscores the statistical robustness of the modeling approach. These MM-adjusted marginal means may serve as valuable population-specific refer-ence intervals for interpreting hematological data in Creole goats managed under extensive systems.

## DISCUSSION

### Hematological variability and contributing factors

The findings of this study revealed substantial inter-individual variability in the hematological profiles of Creole goats managed under extensive production systems, suggesting a multifactorial influence, including environmental, nutritional, and physiological factors, on hematopoiesis and blood cell functionality [24, 25]. Some of this variability was attributable to outliers, parti-cularly among animals fed diets based on stover and alfalfa. These deviations may reflect stress responses linked to physical irritants such as thorns present in agricultural residues like corn or cotton stover, which can cause oral injuries or discomfort [26], thereby affecting specific individuals differently. Although such cases were rare, robust statistical modeling was applied to mitigate their influence and yield representative esti-mates of central tendencies.

### Leukocyte variation and immune status

Elevated coefficients of variation for EOS, MON, and NEU indicate divergent immune responses among individuals. High eosinophil levels are typically associated with allergic reactions or parasitic infections [27], while MON counts rise in the presence of chronic inflam-mation or persistent infections [28]. Neutrophilia is commonly linked to acute bacterial infections [29]. These patterns likely reflect diverse immunological exposures, age-related immunocompetence, and differing health statuses, even within a relatively homogeneous population in terms of breed, diet, and husbandry [30, 31]. Prior studies by Tsheole and Mwanza [31] and Shamsaddini Bafti and Mozaffari [32] have validated the utility of hematological indices as reliable markers of immune stressors, including nutritional deficiencies and para-sitism. The elevated WBC counts observed in this study align with data from Italian goat breeds [13], though they exceed those reported for Tswana goats [33].

### Erythrocyte stability and adaptive hematological responses

In contrast to leukocyte indices, the limited varia-tion observed in MCH and MCV values is consistent with reference ranges reported for clinically healthy goats [13]. The strong positive correlations among HCT, RBC count, and HGB concentration highlight their synergistic role in oxygen transport [34]. Furthermore, the inverse correlation between MCV and MCHC may reflect physiological adaptations by Creole goats to optimize oxygen utilization under environmental or metabolic stress [35].

### Geographic influence and environmental homogeneity

Geographic location did not significantly influence hematological parameters. This lack of effect may be attributed to the ecological and managerial uniformity between Independencia and San Clemente. Although environmental variation has been shown to influence physiological responses in goats [6], the geographical proximity and standardized management conditions likely attenuated such differences. Consequently, the hematological values reported here may serve as regional references for similar agroecological zones [36, 37].

### Dietary effects on hematological parameters

Diet emerged as a key factor influencing hemat-ological outcomes. Goats fed a combination of alfalfa and agricultural residues exhibited elevated HGB conce-ntrations, potentially due to the bioactive compounds and micronutrients in these by-products. Agricultural residues may provide higher iron levels essential for hemoglobin synthesis as well as antioxidant flavonoids and anthocyanins that stabilize erythrocyte membranes and inhibit oxidative hemolysis [17, 18]. Legumes such as beans further contribute amino acids (e.g., histidine) involved in hemoglobin formation [38]. These findings are supported by studies reporting HGB enhancement with red corn supplementation [39] and dried atom leaf inclusion [40].

### Age-related hematological adaptations

MCHC values were significantly higher in 2-year-old goats compared to older individuals, suggesting age-related hematological adaptations [41]. Similar trends have been reported in alpacas, where MCHC declines with age [42]. These observations may reflect developmental differences in immune function, eryth-rocyte turnover, and metabolic demands. Younger goats likely exhibit heightened hematopoietic activity due to the ongoing maturation of their immune system, increased cytokine production, and rapid erythrocyte regeneration, which contributes to elevated hemoglobin content [43, 44].

### BCS as a diagnostic indicator

BCS was a significant determinant of hematol-ogical variation. Lower BCS was associated with reduced RBC and HGB levels, indicative of compro-mised erythropoiesis, which may be caused by chronic undernutrition, iron deficiency, or impaired nutrient absorption [45, 46]. HCT, RBC, and HGB values consistently declined with decreasing BCS, undersc-oring the importance of body reserves in maintaining hematological function [47]. Additionally, poor BCS may indicate chronic energy deficits that impact bone marrow function. Subclinical parasitism, despite rou-tine deworming, may also contribute to anemia and leukocytosis through low-grade inflammation and inte-rmittent blood loss.

Notably, some studies have reported contrasting trends. Torres-Chable *et al*. [48] observed a non-significant inverse association between BCS and hem-atological indices, possibly due to variations in parasitic load or feed quality. Similarly, lower WBC counts have been observed in animals with BCS ≥2.5 relative to those with BCS = 1 [49], as also reported in South American camelids [42]. These differences may stem from immun-ometabolic dysregulation, where poor-condition ani-mals develop hyperactive immune responses due to chronic antigenic exposure, potentially resulting in immunosuppression over time. Thus, BCS serves not only as a nutritional indicator but also as a practical proxy for assessing hematological and health status. Conditions such as fascioliasis or persistent bacterial infections could explain leukocytosis and reduced body condition [47, 50].

### Comparative hematological ranges and breed-specific considerations

Compared to the Argentata dell’Etna breed, RBC, HGB, HCT, and MCHC values in Creole goats were 26%–45% lower [13]. However, values for MCV, MCH, and WBC were consistent across breeds, reflecting the relative stability of these indices. These discrepancies may reflect underlying breed-specific or environmental adaptations. Future research should investigate hema-tological traits in Peruvian Creole goats across diverse geographic regions and compare them with those of other breeds to refine reference intervals and diagnostic interpretations.

### Limitations and recommendations for future research

The hematological parameters obtained in this study were collected exclusively during spring, a sea-son known to influence physiological responses [51]. While differences between locations were noted, their limited magnitude likely stemmed from geographic proximity and similar feeding regimens. Moreover, the modest sample size constrains the generalizability of the findings. Future research should incorporate broader geographic contrasts, seasonal variation, and sex-based comparisons. In addition, the predictive models developed herein should be validated in larger, independent populations to confirm their robustness and utility for field applications.

## CONCLUSION

This study provides a comprehensive characte-rization of hematological parameters in Creole goats reared under extensive production systems on the southern coast of Peru. Notable interindividual variat-ion was observed, particularly in leukocyte indices (e.g., EOS, MON, and NEU), which exhibited coefficients of variation exceeding 50%, indicative of diverse immunological responses within the population. In contrast, erythrocyte parameters such as MCH and MCV demonstrated low variability, consistent with physiological stability in healthy animals.

Key predictors of hematological variability inclu-ded feeding regimen, age, and BCS, while geographic location exerted no statistically significant effect. Speci-fically, goats fed alfalfa combined with agricultural residues exhibited increased HGB levels and reduced MCV, likely due to the synergistic influence of iron-rich and antioxidant compounds in crop residues. Younger animals demonstrated elevated MCHC, suggesting age-related hematological adaptation. In addition, animals with lower BCS consistently exhibited reduced RBC, HGB, and HCT values, underscoring the role of nutritional status in erythropoietic function.

From a practical standpoint, the MM-estimated marginal means derived from robust regression models, adjusted for a standard BCS of 2.7, offer context-specific reference values for hematological interpretation in Creole goats. These values may assist veterinarians and producers in diagnosing subclinical conditions, moni- toring nutritional adequacy, and improving health mana- gement strategies under extensive systems.

The principal strength of this study lies in its use of robust statistical modeling (MM-estimation), which accommodates outliers and non-normal distributions, thereby enhancing the reliability of reference intervals in field conditions. Moreover, the integration of ecological, physiological, and nutritional variables into predictive models enhances the interpretative value of hematological assessments for local production contexts.

In conclusion, this study establishes a foundational hematological reference framework for Creole goats in coastal Peru and highlights the diagnostic value of BCS, age, and diet. Future research should extend this work across seasons, include male animals, and encompass a broader geographic scope to refine and validate these findings. The predictive models developed in this study represent a valuable tool for enhancing herd health and productivity in low-input goat production systems.

## AUTHORS’ CONTRIBUTIONS

JS: Conceptualized and designed the study, developed the methodology, conducted sampling, and drafted the manuscript. EAS and JT: Designed the study, collected samples, and participated in the initial manuscript preparation. PS and MJCC: Assisted in the methodology, sample collection and analysis, prelim-inary results, and reviewed the manuscript. JAR and JCL: Data management, and initial manuscript preparation. JCG and DJC: Contributed to data management and formal analysis, results interpretation, and writing. All authors have read and approved the final manuscript.
